# Practice of emergency obstetric care signal functions and reasons for non-provision among health centers and hospitals in Lake and Western zones of Tanzania

**DOI:** 10.1186/s12913-018-3685-6

**Published:** 2018-12-05

**Authors:** Edward Maswanya, Projestine Muganyizi, Stella Kilima, Deus Mogella, Julius Massaga

**Affiliations:** 1 National Institute for Medical Research (NIMR)-Headquarters, PO Box 9356, Dar-es-Salaam, Tanzania; 2 Muhimbili University of Health and Allied Sciences (MUHAS), PO Box 65001, Dar-es-Salaam, Tanzania; 3 National Blood Transfusion Unit, Ministry of Health, Social Development, Gender, Elderly and Children, PO Box 65019, Dar-es-Salaam, Tanzania

**Keywords:** Obstetric care, Neonatal care, EmOC indicators, Tanzania

## Abstract

**Background:**

The Lake and Western Zones of Tanzania that encompass eight regions namely; Kagera, Geita, Simiyu, Shinyanga, Mwanza, Mara Tabora and Kigoma have consistently been reported with the poorest Maternal Newborn and Child Health (MNCH) indicators in the country. This study sought to establish the provision of Emergency Obstetric Care (EmOC) signal functions and reasons for the failure to do so among health centers and hospitals in the two zones.

**Methods:**

All the 261 public and private hospitals and health centers providing Obstetric Care services in Lake and Western Zones were surveyed in 2014. Data were collected using questionnaires adapted from the Averting Maternal Deaths and Disabilities (AMDD) tool to assess EmOC indicators. Managers in all facilities were interviewed and services, medicines and equipment were observed. Spatial Mapping was done using a calibrated Global Positioning System (GPS) Essential Software for Android and coordinates represented on digitalized map with Arc Geographical Information System (GIS) software. Population data were according to the 2012 Housing and Population National Census.

**Results:**

In total 261 health facilities were identified as providers of Obstetric care services, including 69 hospitals and 192 health centres which constitute an overall facility density of 8 per 500,000 population. The three most common EmOC signal functions available in the 3 months preceding the survey were oxytocics (95.7%), injectable antibiotics (88.9%) and basic newborn resuscitation (83.4%). The lowest proportions of facilities performed Cesarean section (25.7%) and blood transfusion (34.6%). Policy restrictions were the most frequent reasons given in relation to nonperformance of blood transfusion and Cesarean section when needed. Lack of training and supplies were the most common reasons for non availability of assisted vaginal delivery and uterine evacuation. Overall the Direct Case fatality Rate for direct obstetric causes was 3%. The referral system highly depended on hired or shared ambulance.

**Conclusion:**

The provision of EmOC signal functions in Lake and Western zones of Tanzania is inconsistent, being mainly compromised by policy restrictions, lack of supplies and professional development, and by operating under lowly developed referral services.

## Background

The burden of maternal mortality and morbidity is especially high in Africa. Every year over 600,000 women die due to pregnancy-related complications [1, 2]. In Tanzania, maternal mortality ratio is currently at 556 per 100,000 live births and if nothing is done, naturally there will be around 1000 to 1500 deaths per 100,000 births [2–4] . While causes of maternal mortality are mostly known it is acknowledgeable that most deaths could be averted with adequate and timely emergency obstetric care [2, 5].

Monitoring maternal health has therefore moved away from impact measures towards process measures as an accepted proxy [6, 7]. To address this issue a set of standard process indicators has been adopted internationally and used in various surveys [7, 8]. In agreement with this direction, Tanzania’s one plan II strategy (2016–2020) insists on implementing actions and focus resources where interventions will be most effective in averting maternal and childhood deaths [9]. However, unavailability of Emergency Obstetric Care (EmOC) and Reproductive health services has been a challenge.

It has been consistently shown in various studies that the Lake and Western zone regions especially Mara, Mwanza, Shinyanga, Geita, Simiyu, Kigoma and Tabora have been left out with a big gap in many EmOC and Reproductive health indicators [3, 5, 10–14]. Moreover, reports by the Ministry of Health Community Development Gender, Elderly and Children (MOHCDGEC) indicate a serious Human Resource for Health shortage in the country but being the poorest in all the regions of Lake and Western zones [12, 15]. Apart from poor Human Resource for Health, a recent National survey has also reported the two zones as among three zones in the country with the lowest obstetric care facility densities in Tanzania Mainland with all the regions having lower obstetric care facility densities than the National average of 60 per 500,000 population [11, 13, 14]. Accordingly, the National policy under the Sharpened One Plan (2014–2015) had a special focus to address the Geographical inequity for women and children from the two zones [5, 9]. Thus, the focus of the study was to establish the baseline status of Emergency Obstetric Care (EmOC) in the Lake and Western Zones and reasons for failure to provide EmOC services.

## Methods

This Cross-sectional survey was conducted in all the 8 regions of the Lake and Western zones of Tanzania in 2014. Lake Zone is made up of Mara, Geita, Simiyu, Shinyanga, Mwanza and Kagera regions and the Western zone consists of Kigoma and Tabora regions. According to the Tanzania’s National Population and Housing census of 2012 these two zones had a total population of 16,252, 410 and an area of 233, 837 km ^2^. The study collected information at the health facility level using a standard EmOC tool which was developed and used by AMDD to assess the availability, use and quality of emergency obstetrics care [8]. For the purpose of mapping, a calibrated Global Positioning System (GPS) Essential Software for Android was used and coordinates represented on digitalized map using Arc Geographical Information System (GIS) software. Although all levels of health facilities were included in the survey, the current analysis is confined to all health centers and hospitals that provide obstetric care services in the two zones including public and private owned facilities. These two types of health facilities belong to the domain of health facilities that are eligible for provision of Comprehensive Emergency Obstetric Care services in Tanzania.

The survey primarily aimed at assessing all the original six EmOC indicators [8] in the two study zones whose analysis is still ongoing. The current sub-analysis is limited to Geographical distribution of Obstetric Care health facilities specifically for Health Centres and Hospitals, availability of Emergency Obstetric Care signal functions, and the Direct Case Fatality Rate. Data were obtained from interviews with key people in facilities, data reviews and through direct observation as has been recommended by others [8]. Since interviews with Managers and Unit in-charges was about facility information, only verbal consent was considered necessary after the permission to obtain such data was given by the MOHCDGEC. All data were entered in an electronic questionnaire using android tablets and promptly sent to our central database. In order to ensure quality, a technical committee was formed by the MOHCDGEC which supervised the research process. Throughout the survey access to the data was restricted to the research team. Ethical approval for the study was issued by the NIMR Institutional Research Board. We obtained permission to conduct the study from the relevant local authorities.

## Results

### Distribution of obstetric care health facilities

**Fig. 1:**
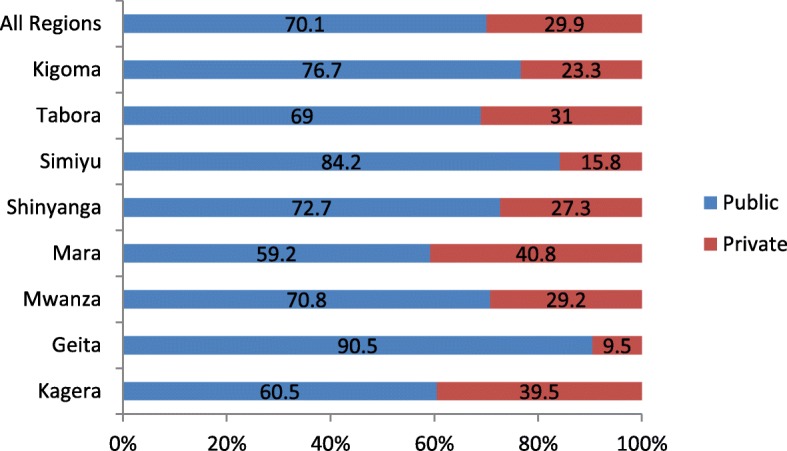
Distribution of health facilities by ownership

Of the total 261 health facilities in Lake and Western zones, 70.1% (183) were public and the remaining 29.9% (78) were private including the faith-based owned. Of all the regions, Mara 40.8% (20) and Kagera 39.5% (17) had the highest proportions of private owned facilities while Geita 9.5%(2) and Simiyu 15.8% (3) had the lowest proportion of Private facilities (Fig. 1).

### Provision of EmOC signal functions

Overall the Obstetric Care facility density in relation to Health Centers and hospital in the Lake and Western zones combined was 8 per 500,000population. Mara region had the highest obstetric care density (14 facilities per 500,000population) of all the regions in the two zones while Geita and Simiyu had the lowest density of 6 facilities per 500,000 population each (Table 1).

**Table 1:** Distribution of obstetric care Health Centres and Hospitals in Lake and Western Zones, Tanzania

Zone	Population	Hospitals		Health Centres		All	Obstetric Care Facility per 500,000popn
		*n*	%	*n*	%		
Lake	11,832,857	53	26.2	149	73.8	202	8.5
Western	4,419,553	16	27.1	43	72.9	59	6.7
Region							
Kagera	2,458,023	15	35	28	65	43	8.7
Geita	1,739,530	4	19	17	81	21	6.0
Mwanza	2,772,509	13	27	35	73	48	8.7
Mara	1,743,830	12	24	37	76	49	14.0
Shinyanga	1,534,808	4	18	18	82	22	7.2
Simiyu	1,584,157	5	26	14	74	19	6.0
Tabora	2,291,623	10	34	19	66	29	6.3
Kigoma	2,127,930	6	20	24	80	30	7.0
Total	16,252,410	69	26	192	74	261	8.0

The provision of EmOC signal functions in the last three months is highly contingent on the availability of patients who need the service. Table 2 shows the percentage of facilities that provided EmOC signal functions after excluding those facilities that did not perform each signal function due to the lack of patients with the indication. Overall provision of uterotonic was the most frequently practiced EmOC signal functions by 96.1% (247) of 257 facilities that attended patients with the indication. Administration of parenteral antibiotics 90.6%(183/202), essential resuscitation of the Newborn 83.0%(191/230), administration of parenteral anticonvulsant 79.8%(130/163) and Manual Removal of placenta 77.2% (139/180), were other most frequently performed functions. Overall CEmOC signal functions of Blood transfusion 48.2% (53/110) and Cesaeran section 45.5% (50/110) were the least provided. Facilities in Kagera region were relatively the most frequent providers of Blood transfusion 81.8%(9/11) and Cesarean section 69.2% (9/13). Private facilities were better than Public facilities in provision of almost all EmOC signal functions.

**Table 2:** Percentage of facilities that performed EmONC signal functions in last three months in presence of patients with the indication

Zone^a^	Injected antibiotics (*N* = 202)	Injected Uterotonics (*N* = 257)	Injected anticonvul sant (*N* = 163)	Manually removed of placenta (*N* = 180)	Evacuated products of conception (*N* = 183)	Assisted Vaginal delivery (*N* = 162)	Resuscit ated a Newborn (*N* = 230)	Blood transfusion (*N* = 110)	Performed Cesarean section (*N* = 110)
Lake	91.4	95.4	78.5	78.9	59.1	41.5	82.4	46.8	46.8
Western	88.2	98.3	83.3	72.3	65.2	28.2	85.2	51.5	41.9
Region^a^									
Kagera	96.6	95.3	73.1	72.7	52.0	64.0	97.4	81.8	69.2
Geita	100	100	85.7	77.8	41.2	35.3	88.9	66.7	57.1
Mwanza	82.5	97.9	62.1	67.6	56.8	30.0	82.6	36.4	39.1
Mara	92.1	89.6	85.7	93.8	78.1	41.4	97.4	54.5	52.2
Shinyanga	94.4	100.0	100	80	58.8	28.6	52.4	18.2	25.0
Simiyu	92.9	94.4	77.8	83.3	55.6	50.0	35.7	20.0	20.0
Tabora	95.7	96.6	100	100	73.7	42.9	75	30.8	35.7
Kigoma	82.1	100.0	70.8	53.6	59.3	20.0	93.3	65.0	47.1
Facility Ownership^a^									
Public	90.0	95.6	78.6	77.9	58.0	33.3	81.2	43.1	42.0
Private	92.0	97.4	82.4	75.5	67.3	51.1	87.1	57.9	51.2
Facility Type^a^									
Hospital	88.9	98.5	77.6	70.2	66.0	47.6	85.7	62.2	60.5
Health Center	91.2	95.3	80.7	79.7	58.6	35.0	82.0	41.1	37.5
Overall ^b^	90.6	96.1	79.8	77.2	60.7	38.3	83.0	48.2	45.5

^a^Within group percentages for facilities that received patients in need of specific signal function in last 3 months; ^b^ Percentages calculated based on all facilities that received patients in need of the specific signal function in last 3 months

**Fig. 2:**
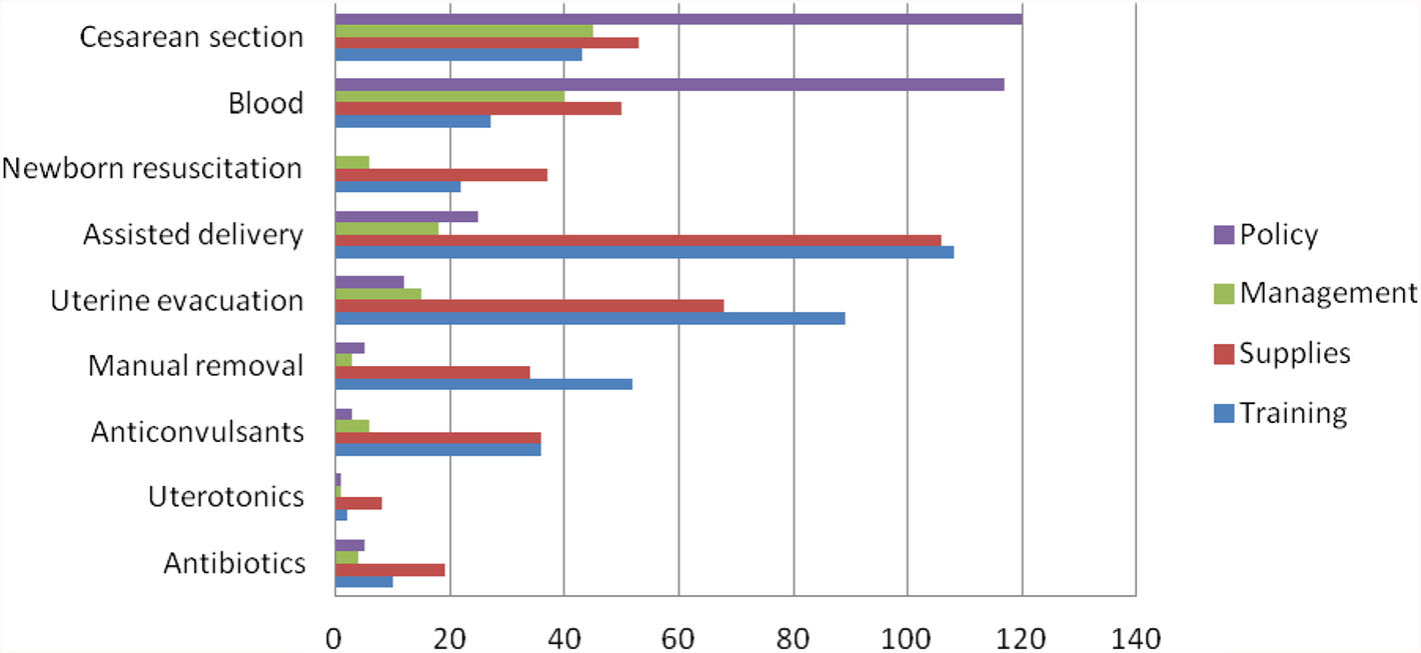
Frequency of reasons given for not providing the various EmOC signal functions when needed

Figure 2 Shows the reasons given for not providing EmOC signal functions in the presence of patients with indications. Policy issues were the reason given by most health facilities for not performing Cesarean Section and Blood transfusion. The lack of supplies and training were the major reasons given for not performing the rest of EmOC signal functions. Conspicuously, the need for training staff and constraints in supplies were most frequently mentioned in relation to Assisted Vaginal delivery and uterine evacuation for the retained products of conception (Fig. 2)

**Fig. 3:**
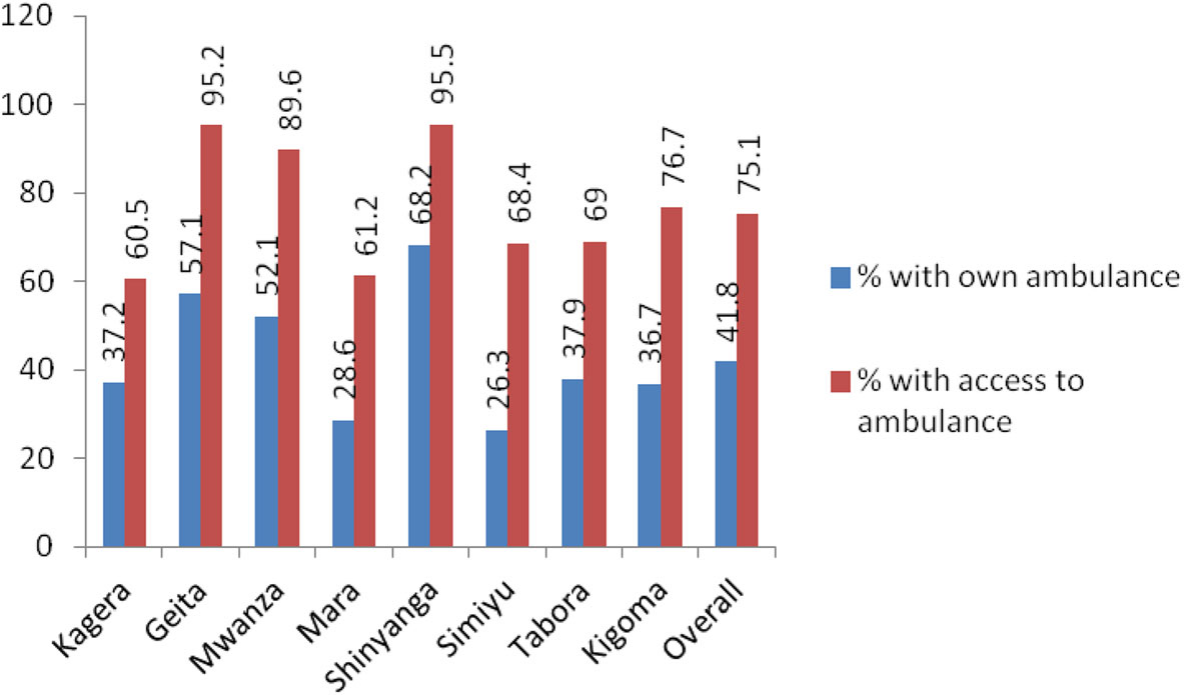
Availability of Ambulance system

In view of the poor availability of EmOC signal functions especially those needing Blood transfusion and Cesarean section in Lake and western zones underscores the need for urgent referral. The availability of ambulance is shown in Fig. 3.

Overall, 75% of hospitals and Health Centre’s reported to be able to access the ambulance but only 41.8% of the facilities owned the ambulance. Most of the interviewed Managers who relied on hired ambulance complained on unreliability of this system.

Direct Case Fatality Rate measures the quality of services provided by health facilities by estimating the number of women who come to facility with direct obstetric complications from which they die. The case fatality rate for all regions in Lake and Western zones considered together was 3%. The highest case fatality rates were reported in Geita (4.8%), Mara (4%) and Simiyu (3.5%.). The lowest Direct Case fatality rate was in Mwanza (1.5%) (Table 3).

**Table 3:** Overall Direct Case Fatality Rates

	Number of maternal death/complications	Case fatality rate (%)
Lake and Western Zones [Population 16,252,410]	718/23,900	3.00
Regions		
Geita	54/1121	4.82
Kagera	104/3280	3.17
Kigoma	79/2522	3.13
Mwanza	66/4306	1.53
Shinyanga	56/2285	2.45
Simiyu	42/1208	3.48
Tabora	115/3944	2.92
Mara	202/5234	3.86

## Discussion

This study of health centers and hospitals in Lake and Western Zones shows an overall Obstetric Care Facility Density (OCFD) of 8 per 500,000 population which is acceptable by international standards, had it been that all these facilities fully implemented EmOC signal functions to which they are entitled. According to International Standards, 5 Obstetric Care facilities per 500,000 population are the needed minimum number if 4 of these had provided the 7 Basic Emergency Obstetric and Care services and at least one had performed Cesarean section and Blood transfusion in last three months [7, 16]. Since all these facilities were entitled to provide all the 9 EmOC signal functions the potential for the two zones to exceed the minimum EmOC requirement is huge, especially considering that dispensaries were not counted in this analysis.

It has also been shown that some variations exist among regions in terms of OCFD and public- private mix of services. Mara, Kagera and Mwanza in the Lake zone had better OCFD and Public private service mix compared with other regions, with Mara almost double the overall OCFD. It was also noted that the new regions of Geita and Simiyu had the lowest OCFD and private-public service mix notwithstanding the quality of care provided by the facilities.

When analyzing the data, it was deliberately decided to consider provision of each EmOC signal function in the context of availability of patients who needed the particular functions in last 3 months. This consideration gives a better impression on the extent to which women who seek EmOC services from hospitals and health centers in the two zones are either likely or not to receive the services. Overall, no EmOC signal function was provided by all the obstetric care facilities in the two zones in last three months. The signal functions that were provided by at least three quarters of the facilities were provision of uterotonic (Oxytocin), parenteral antibiotics, basic resuscitation of the newborn, provision of parenteral anticonvulsant (Magnesium Sulphate) and manual removal of the retained placenta. Only a third or less of facilities was able to provide the life saving services to women who needed assisted vaginal delivery, blood transfusion and Cesarean section. The overall availability of services was better in private than the public facilities. The provision of EmOC signal functions in Lake and Western zones can be compared and contrasted with studies in other developing countries. In Bangladesh, where the OCFD of 8.6 per 500,000 population is comparable with that in current study zones, the provision of Cesarean section (76.5%) and blood transfusion (70.5%) were both much higher compared to the less than half in the current study. Like in Lake and Western Zones, private facilities in Bangladesh performed better than public facilities. Nevertheless, there was relatively lower performance of assisted vaginal delivery (12.2–48.4%) and provision of anticonvulsant (48.6–80.8%) in Bangladesh than in the current study zones [17]. In Zanzibar, all EmOC signal functions were provided by over 70% of hospitals but performance in lower level facilities was poorer with 28% of them partially performing EmOC signal functions or not at all (56%) [18].

EmOC signal functions should be easily available when needed if maternal and newborn morbidity and mortality are to be minimized. Pregnancy complications, such as hemorrhage, are highly unpredictable and can progress rapidly to cause death of the woman and the unborn fetus [19, 20]. In a setting like that of Lake and Western zones where important life saving services such as evacuation of uterus in case of abortion complication or blood transfusion for obstetric hemorrhages are not available to most obstetric care facilities, rapid referral is the remaining option [21]. In the current study, only 41.8% of facilities had their own ambulances. Although overall three quarters of the facilities could access ambulances including the services shared by other hospitals, facility managers were generally concerned with the unreliability of shared ambulances, which leads to excessive delay. The unavailability of reliable referral system in the Lake and Western Zones can inevitably increase maternal and fetal morbidities and deaths.

Provision of full EmOC signal functions in each of the study facilities should have been the most reliable solution for poor maternal and fetal outcomes in the study zones. While all the study facilities had the potential to provide all the 9 EmOC signal functions, policy barriers that restrict the provision of Cesarean section and Blood transfusion to some of the facilities remain the most outstanding reason for nonperformance. Lack of training of healthcare providers and lack of sustainability of supplies, emerged the main reasons for nonperformance of BEmOC signal functions. The government needs to closely focus on these important barriers if EmOC situation in the two zones is to be improved. The ultimate outcome of poor skills due to untrained staff, poor supplies of equipment, medicines and other necessary supplies and poor referral system in the Lake and Western zones are reflected in high Direct Case Fatality Rate of 3.9% which ranges from 1.5% in Mwanza to 4.8% in Geita, with all the rates being higher than the maximum internationally acceptable of 1% [22]. It is also important to note that the highest case fatality rates were observed in the new regions of Geita and Simiyu, which may not have developed strong health systems, hence the need for special attention to these two regions.

The major limitations of this study were the inability to assess all the EmOC process indicators and its reliance on hospital based data although the integration with population data was made for some indicators. Interviews with the women, people in the community and healthcare providers should have given a wider and complete understanding. Nevertheless, the findings of this study are quite useful to inform policy, researchers and program Managers on the status of EmOC process indicators in the two zones. Due to adherence to international standards, the current results can be compared with other results emanating from EmOC studies elsewhere. This study also gives insights on what can be done to improve the situation in the study zones.

## Conclusions

The Lake and Western zones of Tanzania have a good potential to improve EmOC status due to the high Obstetric Care Facility Density. However, the current EmOC performance is generally suboptimal mainly due to policy, training and supplies related barriers.

## Abbreviations

AMDD: Averting Maternal Deaths and Disabilities
BEmOC: Basic Emergency Obstetric Care
CEmOC: Comprehensive Emergency Obstetric Care
EmOC: Emergency Obstetric Care
MRCC: Medical Research Council
MUHAS: Muhimbili University of Health and Allied Sciences
NIMR: National Institute for Medical Research
OCFD: Obstetric Care Facility Density
